# First-Principle Calculations on O-Doped Hexagonal Boron Nitride (H-BN) for Carbon Dioxide (CO_2_) Reduction into C1 Products

**DOI:** 10.3390/molecules29245960

**Published:** 2024-12-17

**Authors:** Guoliang Liu

**Affiliations:** School of Information Technology, Jiangsu Open University, Nanjing 210017, China; liuguol@jsou.edu.cn

**Keywords:** first-principle calculations, monolayer h-BN, O(N) doping, carbon dioxide reduction reaction

## Abstract

With the rapid growth of the world population and economy, the greenhouse effect caused by CO_2_ emissions is becoming more and more serious. To achieve the “two-carbon” goal as soon as possible, the carbon dioxide reduction reaction is one of the most promising strategies due to its economic and environmental friendliness. As an analog of graphene, monolayer h-BN is considered to be a potential catalyst. To systematically and theoretically study the effect of O doping on the CO_2_ reduction catalytic properties of monolayer h-BN, we have perform a series of first-principle calculations in this paper. The structural analysis demonstrates that O preferentially replaces N, leading to decreasing VBM of monolayer h-BN, which is conducive to improving its capability for CO_2_ reduction. The preferential CO_2_ adsorption sites on monolayer h-BN before and after O doping are the N-t site and B-t site, respectively. O doping increases the adsorption strength of CO_2_, which is favorable in the further hydrogenation of CO_2_. During the conversion of CO_2_ into CO and HCOOH via a two-electron pathway and CH_3_OH and CH_4_ via a six-electron pathway, O doping can reduce the energy barrier of the rate determining step (RDS) and change the key steps from uphill reactions to downhill reactions, thus increasing the probability of CO_2_ reduction. In conclusion, O(N)-doped h-BN exhibits the excellent CO_2_ reduction performance and has the potential to be a promising catalyst.

## 1. Introduction

With the growth of the world population, energy shortage and climate change have become important issues the scientific and social concern. Carbon dioxide (CO_2_) emitted by fossil fuels is one of the main factors contributing to global warming. Thus, many countries have proposed “carbon peak” and “carbon neutrality” [1]. To achieve this “two-carbon” goal, reducing CO_2_ emissions and converting CO_2_ to high-value-added chemicals or fuels by using renewable energy sources such as wind or solar are attractive strategies. [2] The carbon dioxide reduction reaction is an important means to achieve this strategy due to its economic and environmental friendliness [3].

Since CO_2_ is a chemically stable molecule and has high C=O bond energy (750 kJ/mol) [4], the choice of catalyst is the key to achieve CO_2_ reduction. Currently, the main developed catalysts for CO_2_ reduction are semiconductors (for instance, TiO_2_, MgO, Ga_2_O_3_, etc.) with a wide bandgap [5,6,7]. Usually, these materials are doped with metal atoms, such as Pt, Cu, Zn, Cr, Pd, and so on [8,9], to improve their catalytic properties. After graphene was discovered by Geim, Novoselov, and co-workers in 2004 [10], two-dimensional (2D) materials have been widely considered. Due to their special dimensional features, 2D materials show excellent catalytic performance for CO_2_ reduction [11], such as transition metal carbides (TMCs) [12], graphitic carbon nitride (g-C_3_N_4_) [13], MXenes [14,15], layered double hydroxides (LDHs) [16], hexagonal boron nitride (h-BN), etc.

Among them, as an analog of graphene, because of its high chemical stability, large specific surface area, and low cost, h-BN has been used as catalyst in the reactions of N_2_ fixation [17], CO oxidation [18], and CO_2_ reduction [19]. However, its application is limited by its wide bandgap. [20,21] Some studies report that single-metal-atom-doped monolayer h-BN possesses excellent performance. [22,23,24,25] In addition, the ability to capture CO_2_ can be improved by modulating the band structure of h-BN by using non-metallic elements, such as P, S, O, F, Cl, C, and so on. [26,27,28] At present, there are few reports about the effect of O doping on the CO_2_ reduction catalytic performance of h-BN.

In this study, to study the catalytic mechanism of CO_2_ reduction by O-doped h-BN, we performed a series of first-principle calculations. Firstly, the O-doping energies for h-BN at different sites were calculated to determine the most possible doping site. Then, the adsorption energies of CO_2_ on the h-BN surface were calculated to obtain the most stable adsorption site. Finally, free energy diagrams for carbon dioxide (CO_2_) reduction into C1 products (including CO, HCOOH, CH_4_ and CH_3_OH) were calculated to analyze the effect of O doping on the speed-limiting step and energy barriers. It is expected to provide theoretical guidance for the development of CO_2_ reduction.

## 2. Results and Discussion

### 2.1. O-Doping Site

**Figure 1:**
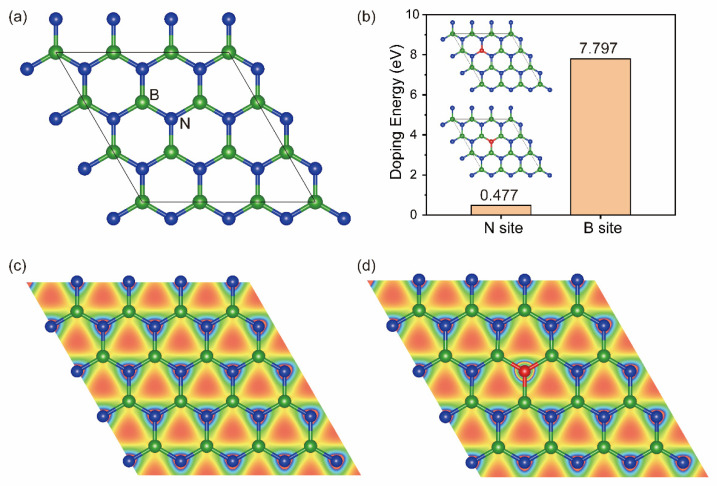
The 3 × 3 supercell of monolayer h-BN, in which the green and blue balls represent B and N atoms, respectively, and the black boxes represent the boundary of the periodic structure (**a**); the doping energy of O substitution for N and B in h-BN, where the O atom is represented by a red ball (**b**); charge density iso-surface plot of monolayer h-BN (**c**) and O(N)-doped h-BN (**d**) (iso-surface value 0.15 e/Å^3^), in which the red and blue regions represent the lowest and highest value of charge density iso-surface.

To study the effect of O doping on the structure of monolayer h-BN, we first constructed a (3 × 3) supercell structure of h-BN, as shown in Figure 1a. Then, a N or B atom was replaced by an O atom. The doping energy of O for N or B was calculated according to the following equation:

(1) $$\Delta E_{doping}=E_{fin}+\frac{1}{n}E_{t}[B\;or\;N_{2}]-E_{ini}-\frac{1}{2}E_{t}[O_{2}]$$

where *E_fin_* and *E_ini_* represent the total energies of h-BN after and before O doping, respectively. *E_t_* is the total energy of B elemental substance or isolated N_2_ or O_2_ gas molecules. The calculated results in Figure 1b show that the doping energy of N replaced by O (0.477 eV) is significantly lower than that of B (7.797 eV), which means that N is preferentially substituted by O. Because the atomic radius of O (r = 0.48 Å) is close to that of N (r = 0.56 Å), the structural deformation caused by O doping can be ignored (as shown in the insert of Figure 1b).

The charge density iso-surface plot in Figure 1c demonstrates that (1) there exists a covalent interaction between B and N, while π electrons are localized around N atoms due to the greater electron affinity of N than that of B; (2) the charge density is dispersed evenly among all the surface atoms. After O doping, we can find from Figure 1d that O atoms connect covalently with the nearest three B atoms through sp^2^ hybridization. The charge around the O atom is rearranged. Because of the stronger electron attraction of O, the electrons of neighboring B and N are more biased toward O.

### 2.2. DOS and Band Structure

**Figure 2:**
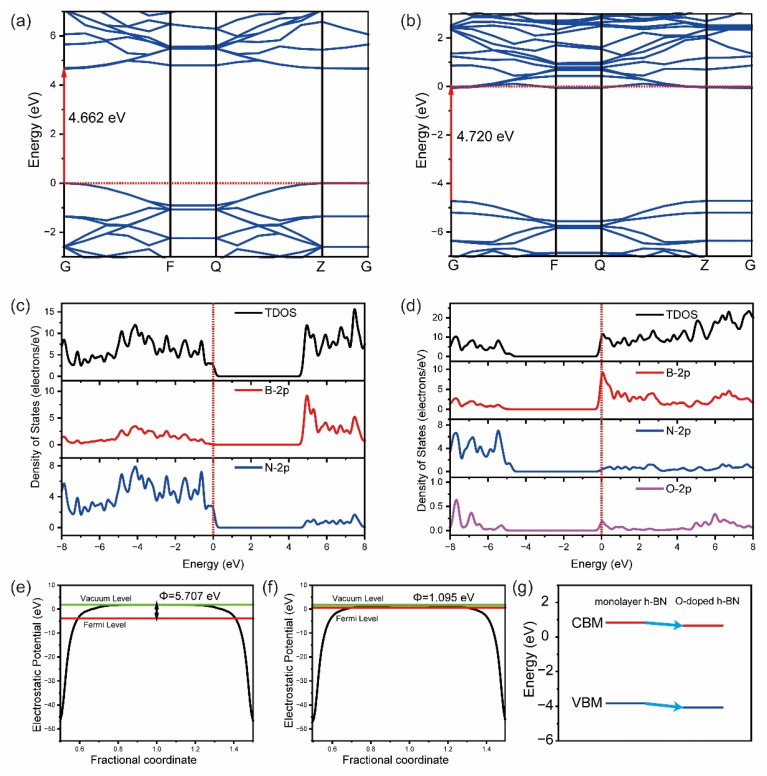
The band structures of monolayer h-BN (**a**) and O(N)-doped h-BN (**b**), in which red arrows indicate the width of the bandgap and the red dotted lines represent Fermi levels; the density of states (DOS) of monolayer h-BN (**c**) and O(N)-doped h-BN (**d**); the work function of monolayer h-BN (**e**) and O(N)-doped h-BN (**f**); and the change in the conduction band minimum (CBM) (red lines) and valance band maximum (VBM) (black lines) for monolayer h-BN and O(N)-doped h-BN (**g**), in which blue arrows show the decrease in the values of CBM and VBM after O doping.

The electronic band structures of monolayer h-BN and O (N)-doped h-BN shown in Figure 2a,b can demonstrate the following interesting results: (1) after O doping, the bottom of the conduction band moves close to the Fermi level, resulting in n-type semiconductor properties; (2) the bandgap is widened from 4.662 eV to 4.720 eV; (3) extra energy bands appear, which originate from excess electrons in O compared to N. These results are further proven by the density of states (DOS) in Figure 2c,d. In addition, the DOS also shows that (1) the electrons of the conduction band are contributed mainly by B 2 p orbitals; (2) there exists the mutual coupling of the 2p orbitals of B and N atoms in the valance band, leading to the strong covalent interaction between B and N for monolayer h-BN; (3) for O(N′)-doped h-BN, because of extra valence electrons supplied from the O atom, a stronger covalent bond between B and O is formed.

Further, as shown in Figure 2e,f, the work functions of monolayer h-BN before and after O doping were calculated. The value of work function Φ is obviously decreased from 5.707 eV to 1.095 eV, which results from the conduction band minimum (CBM) close to the Fermi level after O doping. From Figure 2g, we can find that the value of CBM is decreased from 0.838 eV to 0.658 eV, and the value of the valence band maximum (VBM) is decreased from −3.824 eV to −4.062 eV. Typically, the more positive the CBM is, the stronger the oxidation capacity of the material is, and the more negative the VBM is, the stronger the reduction capacity of the material is. Therefore, the decrease in VBM means that O doping can improve the reduction capacity of h-BN, which is conducive to CO_2_ reduction.

### 2.3. CO_2_ Adsorption

**Figure 3:**
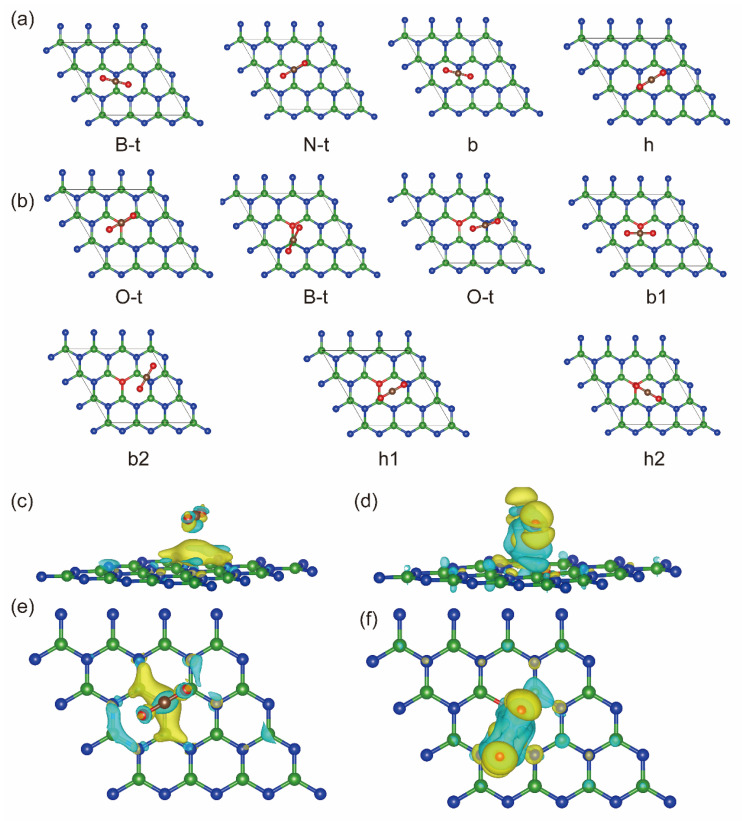
The possible adsorption sites of CO_2_ on monolayer h-BN (**a**) and O (N)-doped h-BN (**b**), in which the green, blue, red and brown balls represent B, N, O and C atoms, respectively. The top view and side views of 3D charge differences of CO_2_ adsorption on monolayer h-BN (**c**,**e**) and O(N)-doped h-BN (**d**), (**f**) (iso-surface value: 0.005 eV/Bohr^3^). In the 3D plot, the yellow and green regions denote charge accumulation and depletion, respectively.

For effective CO_2_ reduction, CO_2_ adsorption is the initial and critical step [29]. Figure 3a shows the adsorption sites of CO_2_ on monolayer h-BN, including (1) the B-t site right above the B atom, (2) the N-t site right above the N atom, (3) the b site between the B and N atoms, and (4) the h site located at the center of the hexatomic ring. After O doping, as shown in Figure 3b, there are three additional adsorption sites: (1) the O-t site right above the O top, (2) the b1 site between the B and O atoms, and (3) the h2 site where two atoms are on the top of the O and B atoms, respectively. To determine the most stable adsorption site of CO_2_, the adsorption energy after structural optimization was calculated according to the following equation:

(2) $$E_{abs}=E_{CO_{2}-surface}-E_{surface}-E_{CO_{2}}$$

where $E_{CO_{2}-surface}$ and $E_{surface}$ represent the total surface energies after CO_2_ adsorption and surface cleaning, respectively. $E_{CO_{2}}$ is the total energy of an isolated CO_2_ molecule. The calculated adsorption energy *E*_abs_, the initial adsorption site, and the final coordinate to which CO_2_ on the surface shifted after relaxation are listed in Table 1 (see Supplementary Materials).

The calculated CO_2_ adsorption energy in Table 1 manifests the following: (1) For monolayer h-BN, CO_2_ molecules can be adsorbed at any site, and the adsorption energy is not much different. However, after O doping, when the initial adsorption sites of CO_2_ molecules are O-t, b1, h1, and h2 sites, the final coordinate of CO_2_ after structural optimization is shifted to the B-t site. This means that O doping results in a decrease in the number of adsorption sites. (2) The preferential CO_2_ adsorption site on monolayer h-BN is the N-t site, while CO_2_ is adsorbed more easily on the B-t site of O(N)-doped h-BN. Meanwhile, after O doping, the adsorption energy of CO_2_ on the BN surface at the optimal site is increased from 0.877 eV to 0.894 eV.

Further, the charge differences of CO_2_ adsorption on monolayer h-BN and O(N)-doped h-BN as shown in Figure 3c–f proved that there exists more obvious electron rearrangement between the CO_2_ and O(N)-doped h-BN surface than in monolayer h-BN. The calculated Bader charge in Table 1 shows that the amount of electrons transferred from the surface to the CO_2_ molecule is increased from 0.345 e to 0.621 e because of O doping for N. This is agreement with the calculated results of adsorption energy.

In summary, O doping for N can increase the adsorption strength of CO_2_ on the h-BN surface but reduces the number of adsorption sites. In addition, the preferential CO_2_ adsorption site on the surface changes from the N-t site to the B-t site.

### 2.4. CO_2_ Conversion to C1 Products

#### 2.4.1. CO_2_ Reduction to CO and HCOOH via a Two-Electron Pathway

CO_2_ can be converted by catalysis into a variety of products, including CO and HCOOH via a two-electron pathway, CH_3_OH and CH_4_ via a six-electron pathway, and other multi-carbon products. At present, the catalytic mechanism of multi-carbon products by using Cu [30] and other materials [31] is relatively complex and not considered in this study. Here, we mainly study the catalytic mechanism of CO_2_ reduction to C1 products.

**Figure 4:**
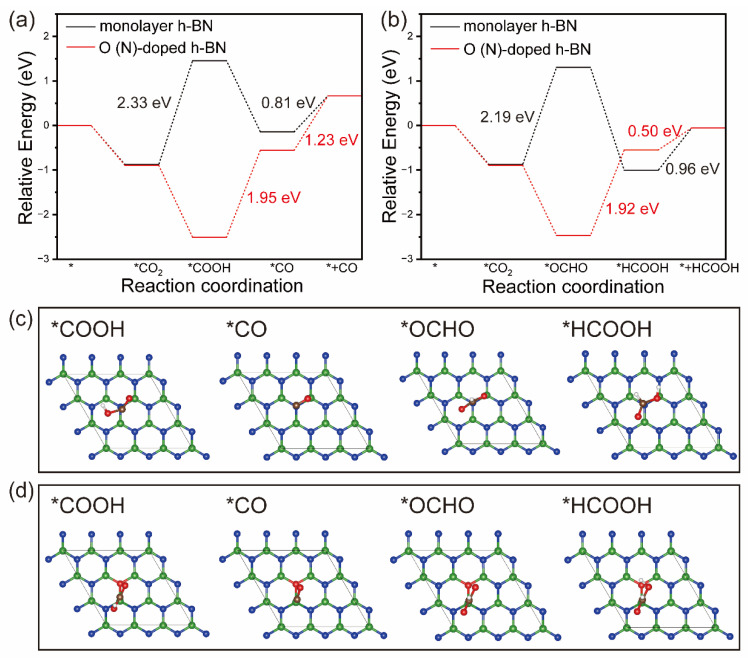
Free energy diagrams for CO_2_ reduction into CO (**a**) and HCOOH (**b**) products on monolayer h-BN and O(N)-doped h-BN, in which the symbol * represents the clean surface. The optimized intermediate adsorption configurations on monolayer h-BN (**c**) and O(N)-doped h-BN (**d**).

As shown in Figure 4a, the CO_2_ reduction to CO tends to proceed along the lines of * → *CO_2_ → *COOH → *CO → CO. The most stable adsorption structures of *COOH and *CO intermediates on the monolayer h-BN surface before and after O doping for N are shown in Figure 4c,d. On monolayer h-BN, CO_2_ need to cross an energy barrier of about 2.33 eV to be activated to *COOH. Then, the proton spontaneously attacks the O of *COOH to form *CO and H_2_O. The final step of CO desorption still needs to overcome the energy barrier of 0.81 eV. However, on O(N)-doped h-BN, the process of CO_2_ reduction to CO is different. CO_2_ can be effectively activated with a reaction energy of −1.62 eV, but the steps of *COOH to *CO and CO desorption are quite difficult at 1.95 eV and 1.23 eV. Comparing the catalytic process on two surfaces, we can find that (1) after O doping, the rate determining step (RDS) changes from *CO_2_ → *COOH to *COOH → *CO, and the energy barrier decreases from 2.33 eV to 1.95 eV; (2) due to strong CO adsorption, CO desorption on O(N)-doped h-BN is more difficult, which facilitates the further hydrogenation of CO.

Figure 4b shows that CO_2_ reduction to HCOOH proceeds along the pathway of * → *CO_2_ → *OCOH → *HCOOH → HCOOH. Figure 4c,d show the most stable adsorption configurations of *OCHO and *HCOOH. On monolayer h-BN, the energy barrier of CO_2_ hydrogenation to *OCHO is 2.19 eV. The second step of *OCHO hydrogenation to *HCOOH is spontaneous. However, on O(N)-doped h-BN, the energy change in the two-step hydrogenation process of *CO_2_ to *HCOOH is the opposite. The energy barrier is 1.92 eV. Further, the reaction energies of HCOOH desorption on two surfaces are 0.50 eV and 0.96 eV, respectively. It can be seen that O doping for N decreases the energy barrier of CO_2_ hydrogenation and HCOOH desorption.

In conclusion, after O doping, the energy barriers of RDS for CO_2_ to CO and HCOOH are reduced; CO desorption becomes more difficult which is conducive to the further hydrogenation reaction, while HCOOH desorption becomes easier. Thus, O doping is favorable in the two-electron catalysis process of CO_2_ reduction.

#### 2.4.2. CO_2_ Reduction to CH_3_OH and CH_4_ via a Six-Electron Pathway

**Figure 5:**
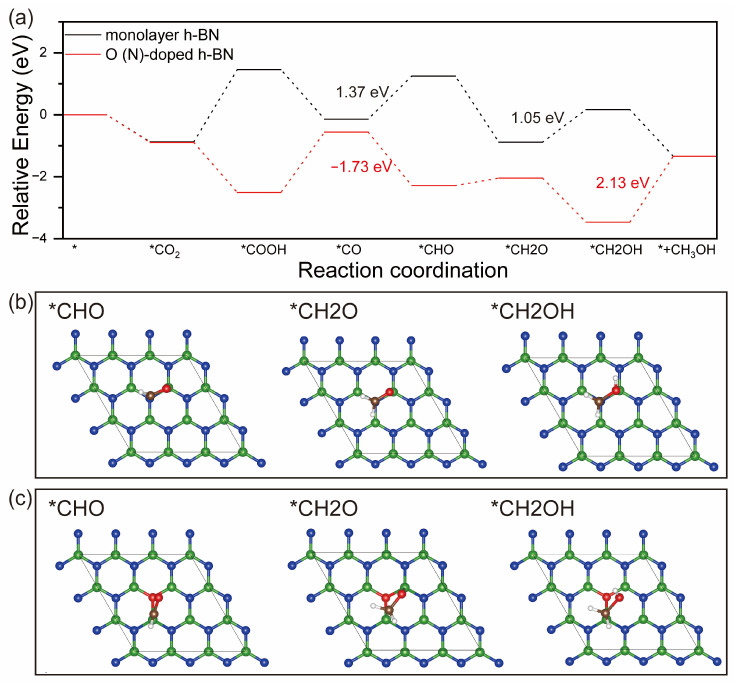
Free energy diagrams for CO_2_ reduction into CH_3_OH (**a**) product on monolayer h-BN and O(N)-doped h-BN, in which the symbol * represents the clean surface. The optimized intermediate adsorption configurations on monolayer h-BN (**b**) and O(N)-doped h-BN (**c**).

The reaction intermediates during the six-electron pathway of CO_2_ reduction to CH_3_OH include *COOH, *CO, *CHO, *CH2O, and *CH2OH, as shown in Figure 5a. The former reduction steps from CO_2_ to *CO are the same as those in the CO production reaction. After the formation of *CO, the residual reaction tends to proceed along the pathway of *CO → *CHO → *CH2O → *CH2OH → CH_3_OH. On the monolayer h-BN surface, the hydrogenation of *CO to *CHO needs to overcome the energy barrier of 1.37 eV, larger than the desorption energy of CO, which means that the further hydrogenation of *CO is difficult, although the reaction of *CHO to CH_3_OH only needs to overcome the lower RDS at an energy of 1.05 eV. In contrast, on the O(N)-doped h-BN surface, the reaction energy of *CO to *CHO is −1.73 eV, which means that the first hydrogenation of *CO can occur spontaneously. After that, the formation of *CH2O is an uphill process at pretty low energy (0.24 eV), while the hydrogenation of *CH2O to form *CH2OH is a downhill reaction with the energy of −1.42 eV. However, the desorption of CH_3_OH on the O(N)-doped h-BN is difficult, with an energy barrier of 2.13 eV. It can be seen from the above analysis that O doping can ensure the further hydrogenation of *CO to CH_3_OH, although it increases the difficulty of CH_3_OH desorption.

**Figure 6:**
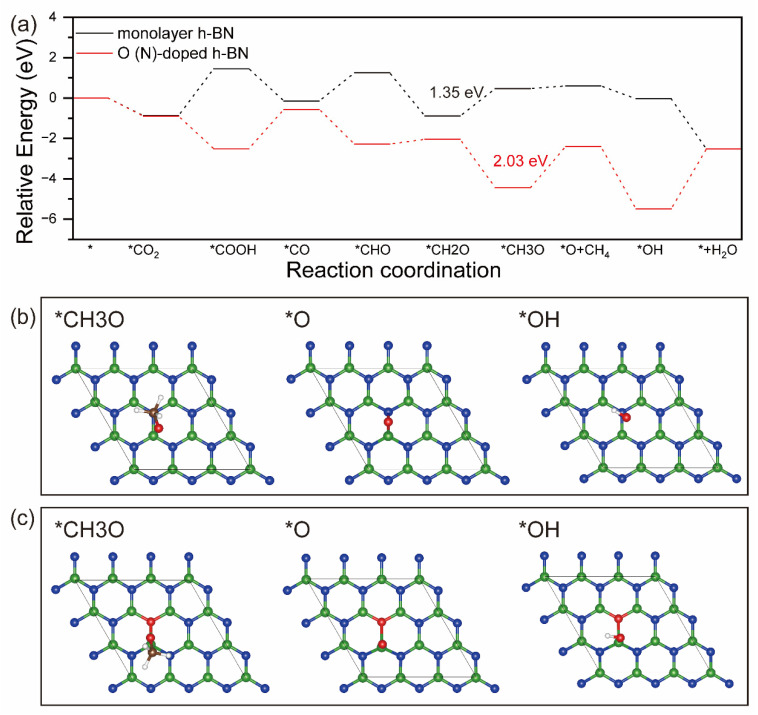
Free energy diagrams for CO_2_ reduction into CH_4_ (**a**) product on monolayer h-BN and O(N)-doped h-BN, in which the symbol * represents the clean surface. The optimized intermediate adsorption configurations on monolayer h-BN (**b**) and O(N)-doped h-BN (**c**).

Figure 6 shows the most favorable reaction pathway for CO_2_ reduction to CH_4_ on monolayer h-BN and O(N)-doped h-BN surfaces, as well as the corresponding optimized intermediate adsorption configurations. By comparing the reaction pathways in Figure 5a and Figure 6a, we can find that the main difference between the formation of CH_3_OH and CH_4_ is the next step of the hydrogenation of CH2O. On monolayer h-BN, after *CH2O is formed, the reaction energy of *CH2O → *CH3O is 1.35 eV, obviously higher than that of *CH2O → *CH2OH with 1.05 eV, which means that the conversion of CO_2_ to CH_4_ is more difficult than CH_3_OH. However, on O(N)-doped h-BN, the reaction of *CH2O → *CH3O is a downhill process, with an energy of −2.39 eV, and lower than that of *CH2O → *CH2OH with −1.42 eV. Meanwhile, the desorption of CH_4_ needs to overcome a lower energy level of 2.03 eV. Thus, after O doping, the formation of CH_4_ is easier than that of CH_3_OH. After CH_4_ desorption, the residual *O can be converted to a H_2_O molecule via a two-electron pathway: *O → *OH → H_2_O.

To sum up, the further hydrogenation reactions of *CO and *CH2O are crucial for the formation of CH_3_OH and CH_4_. Although O doping increases their desorption energies, these two key steps are changed from being uphill reactions to being downhill reactions, leading to the improvement of converting CO_2_ to generate CH_3_OH and CH_4_ via a six-electron pathway.

As a fairly simple way to improve catalytic characteristics, O doping does not reduce the bandgap width of h-BN, but the energy barriers of CO_2_ reduction into C1 products are decreased significantly, especially for the formation of CH_3_OH and CH_4_ via a six-electron pathway. This method can be applied equally as effectively to other catalytic performance improvements of monolayer h-BN, such as N_2_ fixation, NO_3_ reduction, and so on. This provides guidance and reference for the catalytic applications of monolayer h-BN in the future. In this study, the effect of the O-doping concentration has not been investigated, requiring further research in the following works.

## 3. Computational Details

In this paper, all first-principle calculations were performed using Density Functional Theory (DFT) by utilizing the program package DS-PAW (2023B) [32] of Device Studio software (2024A) [33]. We employed the projector augmented wave (PAW) method and the Perdew–Burke–Ernzerhof (PBE) form of the generalized-gradient approximation (GGA) exchange-correlation energy functional. The structural optimizations were carried out by means of the conjugate gradient (CG) algorithm. During structural optimization, all atomic positions were allowed to vary and the lattice parameters were fixed. To accurately calculate the van der Waals force, the DFT-D2 dispersion correction was considered using the Grimme method [34,35]. They would stop until the total energies converged to 10^−6^ eV/atom; the forces on each unconstrained atom were smaller than 0.03 eV/Å. The plane-wave cutoff, *E*_cut_, was chosen to be 400 eV. The k-point mesh of the Brillouin zone was automatically set to 4 × 4 × 1 by using the Gamma Center method. In order to ensure the accuracy of the calculations, the original structure of monolayer h-BN was expanded by 3 × 3 in the x and y directions. In addition, the vacuum thickness in the z direction was set to 10 Å to guarantee sufficient separation between the periodic structures. All model constructions and the subsequent visualizations were performed in the Device Studio software (2024A).

## 4. Conclusions

In this paper, we theoretically investigated the effect of O doping for N on the catalytic mechanism of CO_2_ reduction to C1 products on monolayer h-BN. The following conclusions can be drawn:

1. O preferentially substitutes N of monolayer h-BN, leading to a decrease in the value of VBM, which can improve the reduction capacity of h-BN and is conducive to CO_2_ reduction.
2. The preferential CO_2_ adsorption sites on monolayer h-BN and O(N)-doped h-BN surfaces are the N-t site and B-t site, respectively. O doping increases the adsorption strength of CO_2_ but reduces the number of adsorption sites.
3. After O doping, the energy barriers of RDS during the two-electron conversion reactions of CO_2_ reduction to CO and HCOOH are reduced, but O doping increases the desorption energy of CO, which is conducive to the further hydrogenation reaction of *CO.
4. For CO_2_ reduction to CH_3_OH and CH_4_ via a six-electron pathway, the further hydrogenation reactions of *CO and *CH2O are crucial. O doping can change these two key steps from uphill reactions to downhill reactions, which improves the conversion probability of CO_2_ to CH_3_OH and CH_4_.
5. Our calculations suggest that O(N)-doped h-BN can serve as a promising metal-free CO_2_ reduction catalyst. This theoretical study provides guidance and reference for the future design of high-performance CO_2_ reduction catalysts.

## Figures and Tables

**Table 1:** The calculated CO_2_ adsorption energy *E*_abs_, the initial adsorption site, the final site after structural optimization, and the calculated Bader charge on monolayer h-BN and O(N)-doped h-BN, the “/” represent that there is no change of atomic positions after structural optimization.

System	Initial Adsorption Site	*E*_abs_ (eV)	Shifting to	Bader Charge
monolayer h-BN	B-t	−0.862	/	
	N-t	−0.877	/	0.345 e
	b	−0.867	/	
	h	−0.876	/	
O(N)-doped h-BN	O-t	−0.894	B-t	
	B-t	−0.894	/	0.621 e
	N-t	−0.296	/	
	b1	−0.894	B-t	
	b2	−0.769	/	
	h1	−0.896	B-t	
	h2	−0.895	B-t	

## Data Availability

Research data will be made available on request.

## Supplementary Materials

The following supporting information can be downloaded at: https://www.mdpi.com/article/10.3390/molecules29245960/s1, Figure S1: The K-point and energy cut-off convergence test; Figure S2: The K-path of monolayer h-BN over Brillouin zone; Table S1: The cartesian coordinates of monolayer h-BN and O(N)-doped h-BN after structural optimizations.
