# Supplementary material for: First-Principle Calculations on O-Doped Hexagonal Boron Nitride (H-BN) for Carbon Dioxide (CO2) Reduction into C1 Products
Source: Molecules. 2024 Dec 17;29(24):5960. doi: 10.3390/molecules29245960 (PMC11677389; doi:10.3390/molecules29245960)
Supplement: Supplementary file 1 [file molecules-29-05960-s001.zip › molecules-3362934-supplementary.pdf]

First-Principle Calculations on O-Doped Hexagonal Boron Nitride (H-BN) for Carbon Dioxide (CO_2) Reduction into C1 Products

Guoliang Liu

School of Information Technology, Jiangsu Open University, Nanjing 210017, China; liuguol@jsou.edu.cn

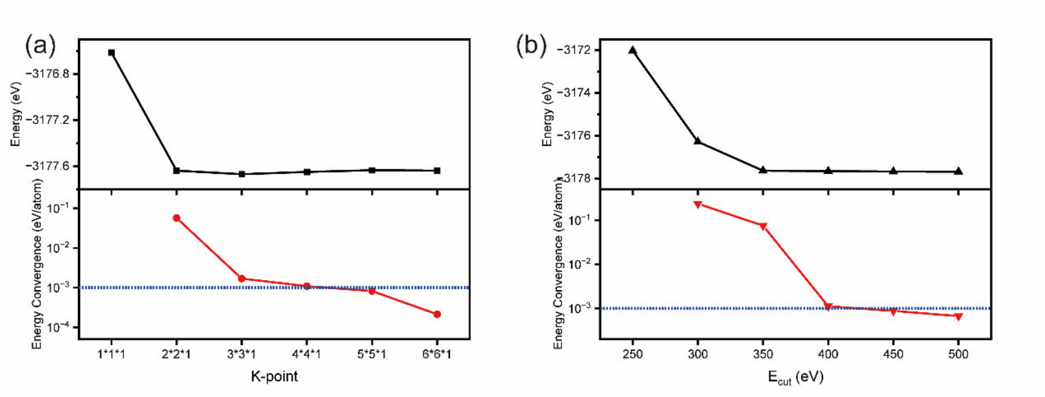

Figure S1. The K-point and energy cut-off convergence test.

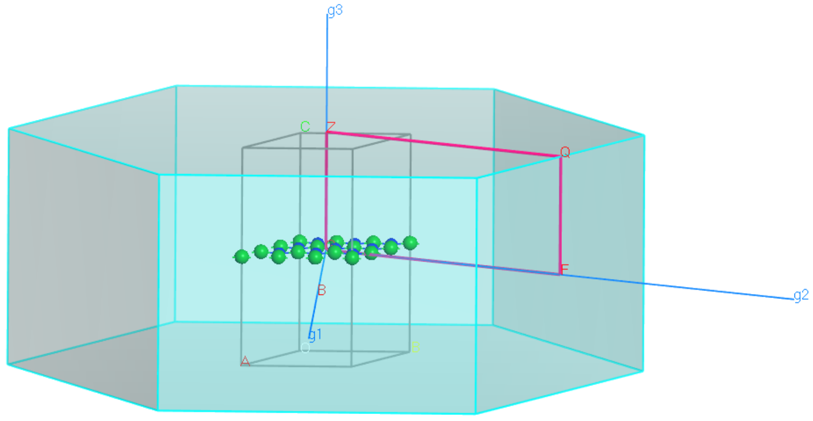

Figure S2. The K-path of monolayer h-BN over Brillouin zone.

Table S1 The cartesian coordinates of monolayer h-BN and O(N)-doped h-BN after structural optimizations.

Atom	Cartesian coordinates of monolayer h-BN	Cartesian coordinates of O(N)-doped h-BN
B1	(0 0 7.5)	(0 0 7.5)
B2	(2.503 0 7.5)	(2.503 0 7.5)
B3	(5.007 0 7.5)	(5.008 0 7.5)
B4	(-1.252 2.168 7.5)	(-1.252 2.163 7.5)
B5	(1.252 2.168 7.5)	(1.252 2.122 7.5)
B6	(3.756 2.168 7.5)	(3.764 2.163 7.5)
B7	(-2.504 4.336 7.5)	(-2.504 4.338 7.5)
B8	(0 4.336 7.5)	(0 4.359 7.5)
B9	(2.504 4.336 7.5)	(2.544 4.360 7.5)
N1	(0 1.445 7.5)	(0 1.452 7.5)
N2	(2.504 1.445 7.5)	(2.503 1.452 7.5)
N3	(5.007 1.445 7.5)	(5.008 1.445 7.5)
N4	(-1.252 3.614 7.5)	(-1.246 3.611 7.5)
N5 (O)	(1.251 3.614 7.5)	(1.252 3.614 7.5)
N6	(3.756 3.614 7.5)	(3.750 3.611 7.5)
N7	(-2.504 5.782 7.5)	(-2.504 5.782 7.5)
N8	(0 5.782 7.5)	(0.005 5.778 7.5)

N9	(2.504 5.782 7.5)	(2.500 5.779 7.5)

1. Structure analysis

Comparing the cartesian coordinates of monolayer h-BN and O(N)-doped h-BN after structural optimizations in Table S1, we can find that the effect of O replacing N on the structure is very small, and the atomic positions hardly changes. This is mainly because of that the atomic radius of O (r=0.48 Å) is close to that of N (r=0.56 Å).
